# Analysis and Prediction of Sulfate Erosion Damage of Concrete in Service Tunnel Based on ARIMA Model

**DOI:** 10.3390/ma14195904

**Published:** 2021-10-08

**Authors:** Dunwen Liu, Haofei Chen, Yu Tang, Chun Gong, Yinghua Jian, Kunpeng Cao

**Affiliations:** School of Resources and Safety Engineering, Central South University, Changsha 410000, China; dunwen@csu.edu.cn (D.L.); chenhaofei@csu.edu.cn (H.C.); gongchun@csu.edu.cn (C.G.); jyh__0412@csu.edu.cn (Y.J.); 205511007@csu.edu.cn (K.C.)

**Keywords:** sulfate erosion, ARIMA model, service tunnel, Erd prediction, mass prediction

## Abstract

Sulfate erosion is a major cause of concrete durability deteriorations, especially for the service tunnels that suffer sulfate erosion for a long time. Accurately predicting the concrete damage failure under sulfate erosion has been a challenging problem in the evaluation and maintenance of concrete structures. Here we design the dry–wet cycle test of service tunnel concrete under sulfate erosion and analyze the Elastic relative dynamic modulus (Erd) and mass under 35 times cycle periods. Then we develop an autoregressive integrated moving average (ARIMA) prediction model linking damage failure to Erd and mass. The results show that the deterioration of concrete first increased and then decreased with an extension of the dry–wet cycle period. Moreover, based on a finite set of training data, the proposed prediction approach shows high accuracy for the changes of concrete damage failure parameters in or out of the training dataset. The ARIMA method is proven to be feasible and efficient for predicting the concrete damage failure of service tunnels under sulfate erosion for a long time.

## 1. Introduction

With the popularization of concrete material, people began to use concrete material for infrastructure construction on a large scale. Concrete has also become the second most utilized building material after water [1]. Because concrete is widely used in a variety of environments, complex environmental factors will lead to premature failure and instability of concrete structures. Sulphate erosion is the most important factor for controlling the durability of concrete structures in corroded environments [2]. There are a large number of sulfate erosion strata in western China, and these corrosive substances may have corrosive effects on the tunnel lining in service [3]. Ma first reported a case of damage to a concrete structure caused by the form of thiobacite during a 2004 survey of the Babanxia Hydropower Station in western China [4]. In the Chengdu-Kunming Railway, which has been completely built, a large amount of erosion damage was found in the tunnel, which was analyzed as sulfate erosion damage. The physical crystallization caused by ettringite sulfate erosion and expansive ettringite crystallization are the main reasons for spalling and the loss of cementation strength in the concrete surface layer of the railway tunnel lining in southwest China [5]. The prediction of erosion failure of the second lining concrete in the service tunnel under a sulfate erosion environment has become one of the problems that urgently need to be solved in tunnel construction.

Different from sulfate corrosion, sulfate erosion refers to the reaction between a sulfate medium and hydration reactants in concrete to form new hydrates. The newly formed hydrate expands and finally destroys the structure. The most common minerals produced inside sulfate-eroded tunnel concrete are gypsum and ettringite [6]. The evolution of tunnel concrete sulfate damage is often affected by the sulfate concentration [7,8], temperature [9,10,11], humidity [12], concrete cement composition [13,14,15] and fly ash content [16,17] in the tunnel. Due to the complex damage mechanism of concrete under sulfate erosion and numerous influencing factors, many scholars explored the damage failure mechanism of sulfate erosion through various experimental methods to establish a research method for predicting damage failure of concrete under sulfate erosion. Rui He studied the damage mechanism of concrete at the microscopic level, namely the interfacial transition zone, under sulfate erosion in the dry–wet cycle process. The results showed that with the increase in the dry–wet cycle, the porosity of the interfacial transition zone gradually increased, and the sulfate solution could compensate for the microstructure of concrete [18]. Ren proposed a formula for calculating the reaction rate constant of gypsum based on the kinetics theory of the chemical reaction, calculated the macroscopic tensile stress based on the volume fraction of gypsum, predicted the cycle times that the concrete could bear, and then calculated the failure time of the concrete [19]. Based on different soaking times of concrete under sulfate ion concentration distributions and nominal stress–strain curve, Liao adopts the method of innovation for the different contents of sulfate ions and the true stress–strain curve of concrete. This paper proposes a concrete member structure that can predict sulfate erosion performance of the actual constitutive model [20]. Silva immersed self-compacting concrete in 5% Na_2_SO_4_ and MgSO_4_ solutions to evaluate the changes in mass, loss of mechanical strength, and linear expansion, which were reflected in the change in concrete length, loss of mass, and compressive strength [21].

Although many scholars have focused on concrete sulfate erosion and explored it through experimental methods, the prediction of concrete damage failure for in-service tunnels is limited. The ARIMA model was a time series prediction method proposed by Box Jenkins in 1976 that could be applied to small samples [22]. The ARIMA model is an extension of the ARMA model, including the autoregressive (AR) model, the moving average (MA) model, and the Integrated method (I). The ARIMA model is characterized by strong robustness, strong predictability of short time series and ease of application, and is widely used in the prediction of various disciplines [23].

In this article, according to the essence of corrosion, the elasticity relative dynamic modulus (Erd) and the mass of the concrete are taken as the basic indexes of damage and failure of concrete specimens [24,25]. Service tunnel concrete under the dual action of long-term sulfate erosion and a dry–wet cycle was used to prepare specimens. The ultrasonic nondestructive testing method has been widely used in concrete nondestructive testing [26,27,28,29]. Karimaei used ultrasonic pulse velocity (UPV) technology as a nondestructive testing method to estimate the compressive strength of 11 groups of concrete samples containing coal gangue. The compressive strength and UPV parameters of concrete with different ages and different proportions of coarse and fine aggregate replaced by coal gangue were studied. The exponential relationship between compressive strength and UPV was obtained [30]. Marek uses ultrasonic pulse technology to conduct nondestructive testing of concrete defects, and uses a convolutional neural network (CNN) to automatically record defect images in concrete components [31]. Doreen used ultrasonic technology to explore the influence of concrete moisture content on mechanical properties and ultrasonic wave velocity of concrete in order to evaluate the static properties of saturated concrete [32]. In recent years, the ultrasonic pulse has become the focus of attention of many researchers. Previous studies have also established the relationship between UPV and durability indicators such as concrete permeability, porosity, water absorption and fly ash content [17,33,34,35]. However, there is very little literature about the use of ultrasonic nondestructive testing to directly detect the damage degree of concrete in a service tunnel. Therefore, this paper aims to use the ultrasonic nondestructive testing method to measure the damage degree of concrete.

In this article, the Erd of the concrete is calculated by using the tested propagation velocity to reflect the degree of damage inside the concrete through the correlation between the propagation velocity of ultrasonic pulse in concrete and the degree of damage inside the concrete. A three-dimensional hierarchical analysis method was used to process the relevant data and obtain the improved Erd after optimization. The subsequent damage failure time of the prepared service tunnel concrete specimens was predicted using the ARIMA model after the optimization of experimental data. The prediction results can provide some reference for the damage development and damage degree of concrete in a tunnel.

## 2. Materials and Methods

### 2.1. Project Summary

**Figure 1:**
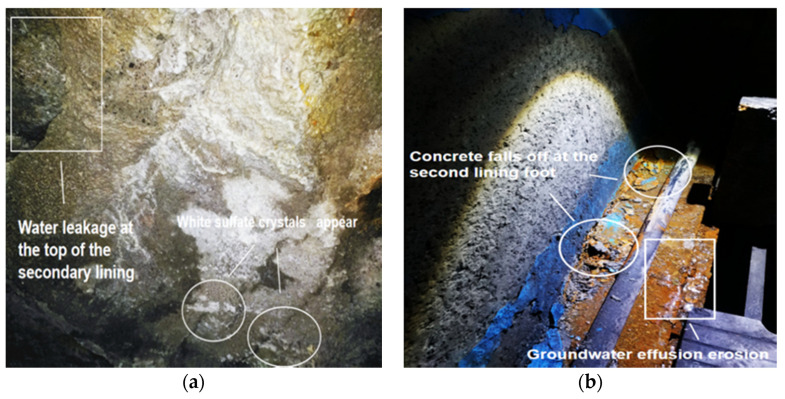
Corrosion of tunnel: (**a**) sulfate crystals precipitated from tunnel concrete; (**b**) collapse and spalling of concrete with secondary lining feet.

This research is based on a service highway tunnel in Chongqing, China. The highway tunnel fully opened in 2006, and it has a design life of 300 years. However, in 2015 and 2018, there were a lot of disasters. As shown in Figure 1, the current situation of expansion and cracking of the secondary lining in the tunnel had been extended to the side wall of the secondary lining.

In Figure 1, with the accumulation of time and long-term infiltration of groundwater, a large number of white sulfate crystals were produced on the secondary lining surface of the tunnel. The tunnel adopts the “New Austrian Method” design principle and is lined with C25 plain concrete sprayed from ordinary Portland cement (OPC). The secondary lining concrete aggregate consists of machine-made sand with a particle size of 0–4.75 mm and continuous-graded gravel with a particle size of 5–25 mm.

### 2.2. Research Program

#### 2.2.1. Test Principle

The change of the Erd of concrete can reflect the deterioration and compactness of the internal structure. When there are holes and cracks in the concrete, the ultrasonic wave will bypass the defects, resulting in an increase in sound propagation. When the concrete is eroded by sulfate, the porosity and compactness inside the concrete specimen are changed, which causes the change of ultrasonic speed [36]. Therefore, the measurement of the ultrasonic speed of concrete specimens can effectively reflect the internal changes of concrete.

The acoustic parameter oscilloscope adopts a transmitting transducer to continuously transmit ultrasonic waves to the concrete. The ultrasonic nondestructive testing device is the acoustic parameter tester HS-CS4EL (The instrument was purchased from Tianhong Electronic Research Institute of Xiangtan city, China) according to the (China Concrete Ultrasonic testing instrument verification regulation for ultrasonic nondestructive testing of concrete (JJG-070-2006)) [37]. The ultrasonic waves continuously propagate between the concrete and the receiving transducer at the other end receives the acoustic signals sent. The received signal is reflected on the oscilloscope by electronic technology, and the received electrical signal is amplified and quantified to obtain the acoustic parameters, such as waveform, acoustic wave speed, amplitude, frequency and propagation time.

#### 2.2.2. Test Materials and Experimental Setup

The tunnel sulfate corrosion of the secondary lining part of the core was made into a 50 mm × 100 mm cylindrical test block. Polycarboxylate high-efficiency water reducing agent (SP) was used as the admixture. The concrete strength and design mix ratio are shown in Table 1. After curing and standing in the mold at a temperature of 20 °C ± 5 °C for 24 h, the mold was then numbered and removed. After the mold was removed, it was immediately put into the standard curing room for maintenance. The temperature was set at 20 °C ± 2 °C, and the humidity RH was 95%.

We used a dry–wet cycle experiment to accelerate sulfate erosion. The degradation mechanism of concrete blocks in the accelerated experiment is consistent with that of concrete in service [18], so it is necessary to select the appropriate concentration of the sulfate solution.

The concentration of sulfate suggested by the Chinese standard (Standard test method for long-term performance and durability of ordinary concrete (GB/T 50082-2009)) is 5% for the evaluation of concrete [38]. Table 2 shows the detection results of underground water samples at the position where concrete is drilled in the tunnel. It can be seen from Table 2 that the sulfate concentration in the water sample from the concrete drilled in the tunnel exceeds 200 mg/L. In some severely corroded areas, the sulfate ion concentration even exceeds 1000 mg/L, such as ZK83+565. According to the standard of (environmental action classification in the Code for Durability Design of Concrete Structures (GB/T 50476-2008)) [39], the corrosion is grade D (severe). However, due to the high sulfate concentration in the water sample detection of the test tunnel, the experiment requires a strict sulfate concentration. Therefore, sodium sulfate immersion concentrations of 2% and 10% are selected, which are also used in previous studies [40,41,42]. To ensure the concentration of the sodium sulfate soaking solution remains unchanged, we replaced it every 30 times. The soaking box used in the experiment is the constant temperature concrete curing box YH-40B, and the drying box is the electric heating air blowing drying box DHG 101-2B (both instruments were purchased from Shanghai Huyue Instrument Equipment Factory, Shanghai, China).

### 2.3. Data Analysis Method

#### 2.3.1. Calculation of Integrated Wave Velocity

As the corrosion aging process inside the concrete is not uniform, there is a difference in the corrosion degree in each direction. In this test, the acoustic velocity in five directions was obtained for each test. We divided the test block into four equal parts and marked it. The circular arc probe was used to measure the transverse sound velocity, and the plane probe was used to measure the longitudinal sound velocity in five directions in total. At present, most acoustic wave tests only measure one direction, and there is a large error, so we use a three-dimensional analytic hierarchy process (TAHP) to improve it by measuring acoustic wave data in multiple directions to correct and reduce the error. The calculation formula is as follows:

(1) $$V_{0}{}^{\prime }=\sqrt{{\left(w_{1}V_{1}{}^{\prime }\right)}^{2}+{\left(w_{2}V_{2}^{\prime }\right)}^{2}+{\left(w_{3}V_{3}{}^{\prime }\right)}^{2}+{\left(w_{4}V_{4}^{\prime }\right)}^{2}+{\left(w_{5}V_{5}^{\prime }\right)}^{2}}$$

where $V_{1}{}^{\prime }$, $V_{2}{}^{\prime }$, $V_{3}{}^{\prime }$, $V_{4}{}^{\prime }$ and $V_{5}{}^{\prime }$ are longitudinal wave velocities in five directions in m/s; $V_{0}{}^{\prime }$ is the comprehensive initial longitudinal wave velocity in m/s; $w_{1}$, $w_{2}$, $w_{3}$, $w_{4}$ and $w_{5}$ are the weights of longitudinal wave velocity in each direction.

#### 2.3.2. Elastic Relative Dynamic Modulus

The Chinese standard(Standard test method for long-term performance and durability of ordinary concrete (GB/T 50082-2009)) stipulates that when the Erd is reduced to 60%, it is judged as the failure of concrete specimen [38]. Because there is liquid in the concrete, the ultrasonic test error will be affected, so the test time was set to two h after the natural heat dissipation after drying. According to Equations (3) and (4), it can be calculated.

(2) $$E_{d}=\frac{\left(1+\mu \right)\left(1-2\mu \right)}{\left(1-\mu \right)}\rho V^{2}$$

(3) $$E_{rd}=\frac{E_{t}}{E_{0}}=\frac{V_{t}{}^{2}}{V_{0}{}^{2}}$$

where $E_{d}$ is the dynamic elastic modulus of the concrete; $E_{t}$ is the dynamic elastic modulus of the concrete at time t; $E_{0}$ is the dynamic elastic modulus of the concrete at time 0; μ is Poisson’s ratio; ρ is the density of the concrete (kg/m^3^); V is the speed of sound during the ultrasonic test; $V_{0}$ and $V_{t}$ are, respectively, the acoustic velocity of the concrete specimen without the dry–wet cycles and the acoustic velocity of the concrete specimen after the dry–wet cycles.

#### 2.3.3. Mass Data

An electronic scale with an inductivity of 0.01 g was used to test the mass loss of concrete after each dry–wet cycle. Referring to the experimental method in the Chinese standard (Standard test method for long-term performance and durability of ordinary concrete (GB/T 50082-2009)), when the mass loss of concrete reaches 5%, the concrete is judged to have failed [38]. The calculation formula for the mass loss of the concrete specimen.

#### 2.3.4. ARIMA Model

The ARIMA model is a model established using the difference method to obtain stationary data from non-stationary data in the ARMA model. It includes the AR model, MA model and Difference (I) method. ARIMA has three characteristics: *p*, *d* and *q*, where *p* is the order of the AR term, *q* is the order of the MA term, and *d* is the difference order required to make the time series stable.

The difference method eliminates the changes on the level of time series, and it is necessary to differentiate the unstable data for the prediction of the ARIMA model. To ensure the effective use of data, the model can extract useful information by a second-order difference at most. Among them, the first-order difference and second-order difference can be expressed mathematically by the equations:

(4) $$\mathrm{First}\;\mathrm{difference}:{y^{\prime }}_{t}=y_{t}{-y}_{t-1}$$

(5) $$\mathrm{Second}\;\mathrm{difference}:{y^{\prime \prime }}_{t}=y_{t}{-2y}_{t-1}+y_{t-2}$$

(6) $$\mathrm{AR}:{\;y}_{t}=c+\alpha_{1}y_{t-1}+\alpha_{2}y_{t-2}+\cdots +\alpha_{p}y_{t-p}+\varepsilon_{t}$$

(7) $$\mathrm{MA}:{\;y}_{t}=c+\varepsilon_{t}+\theta_{1}\varepsilon_{t-1}+\theta_{2}\varepsilon_{t-2}+\ldots \ldots +\theta_{p}\varepsilon_{t-p}$$

(8) $$\mathrm{ARIMA}\;:{\;y}_{t}=c+\alpha_{1}y_{t-1}+\alpha_{2}y_{t-2}+\cdots +\alpha_{p}y_{t-p}+\varepsilon_{t}+\theta_{1}\varepsilon_{t-1}+\theta_{2}\varepsilon_{t-2}+\cdots +\theta_{q}\varepsilon_{t-q}$$

where $y_{t}$ is the non-stationary time series data and the observed value of the time stamp. $y_{t-i}$ is the past time series value; $y_{t-2}$ is the observed value of the time stamp; $y_{t}^{\prime }$ is the time series after the first-order difference of the non-stationary time series; $y_{t}^{\prime \prime }$ is the time series after the second-order difference; c is the intercept constant term; $\alpha_{i}$ is the autoregressive average model coefficient; $\theta_{i}$ is the moving average model coefficient; and $\varepsilon_{t}$ is the white noise process with the variance of $\sigma^{2}$.

**Figure 2:**
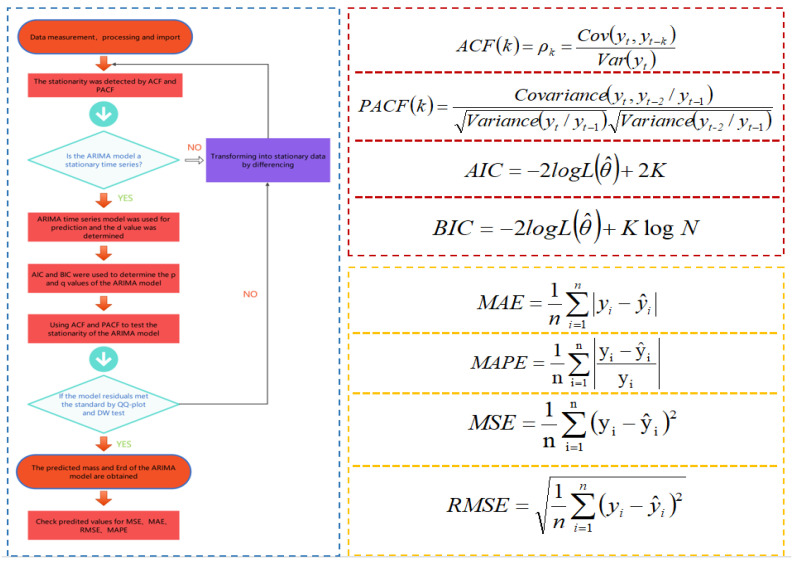
Algorithm showing the methodology for developing ARIMA models.

Figure 2 shows the calculation frame diagram of the ARIMA model established by computer deep learning modeling software, which can obtain a fairly stable time series prediction model of ARIMA mass and Erd according to the specific calculation framework. The lag *k* refers to the correlation between the observed data with an interval of *k* time periods. In the expression of the PACF function, *k* = 2 is taken. $\log\;L\left(\hat{\theta }\right)$ is the likelihood function; *K* is the total number of model parameters; *N* is the number of observations; ${\hat{y}}_{i}$ is the model’s predicted value; $y_{i}$ is the actual value. The established regression model was evaluated according to MAE, MSE and RMSE.

##### Test of Model Data: ACF and PACF

The test of data stability is an important step in time series analysis. The autocorrelation function (ACF) and partial autocorrelation function (PACF) of the autocorrelation graph are used to test the data stability. ACF is the correlation between the former time series data and the present time series data. It describes the linear relationship between the observed values at the moment *t* and the moment (*t* − *k*). The PACF eliminated the influence of other random variables. It simply measured the correlation between time series data and lag value.

##### Fitting Model Data: AIC and BIC

The minimum criterion method of Akagi Information Criterion (AIC) and Bayesian Information Criterion (BIC) was used to determine the order *p* and *q* of the model. AIC based on the concept of entropy is a standard to measure the optimal fitness of statistical models. It encourages the optimal fitness of data fitting but tries to avoid overfitting. BIC is a basic method in statistical pattern recognition. Both AIC and BIC used the penalty likelihood criterion. Compared with AIC and BIC, BIC imposes a greater penalty on the model. Thus, BIC will obtain a more simplified model.

##### Residual Test of the Model: QQ-Plot and D–W Test

The ARIMA model needs to carry out a residual test to ensure that the order is appropriate. The residual test includes a QQ-plot (quantile-quantile plot) test and a Dubin–Watson test. The QQ-plot test is used to intuitively verify whether a set of data is normally distributed. If the sample data deviates too much from the line, an unreasonable model will be shown. The Dubin–Watson test is the test statistic of the residual autocorrelation of the diagnostic model proposed by Dubin and Waston.

##### Error Estimation

The prediction accuracy is evaluated by the mean absolute error (MAE), mean absolute percentage error (MAPE), mean square error (MSE) and root mean square error (RMSE).

### 2.4. Test Procedure

**Figure 3:**
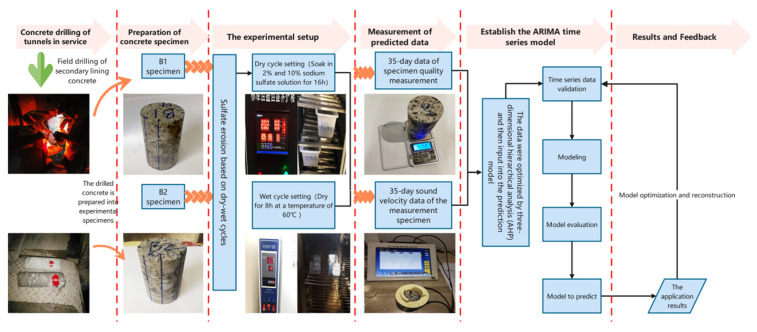
Schematic diagram of experiment and prediction process.

Figure 3 shows the test and prediction process scheme diagram. The test procedure is as follows:

(1) The concrete samples we used were drilled from an in-service tunnel that had been eroded for some time. The prepared concrete specimens were numbered B1 and B2. They were drilled from different parts of the tunnel. Their internal erosion is different, so B1 and B2 are two completely independent concrete specimens. Then, we put them into the designed sulfate solution. The sulfate corrosion was accelerated by dry–wet cycles. The cycle period is 24 h, of which 16 h is the soaking period. We put it into the soaking box with 2% and 10% sodium sulfate solution to ensure that the concrete specimens were fully soaked. The temperature was set at 20 °C, and the relative humidity was 95%. After soaking for 16 h, the concrete specimens were taken out and put into the electric air drying box for drying, and the drying temperature was set at 60 °C.

(2) Then, we took the specimens out and placed them in a ventilated place to cool to room temperature. The four directions and axial directions from the concrete test block’s side were taken for ultrasonic nondestructive testing. The first wave method was used to measure the longitudinal ultrasonic speed of the concrete specimen during the ultrasonic nondestructive testing. To ensure the accuracy of experimental data and reduce the systematic errors generated in the instrument, experimental environment and first-wave pickup, the plexiglass test block with the same size as the specimen was used as the calibration test block during the test in this experiment [37]. When analyzing the change in the specimen’s ultrasonic speed, the difference between the actual ultrasonic speed measured by the specimen and the ultrasonic speed measured by the calibration test block on the same date was used as the analysis object.

(9) $$\Delta v=v_{\mathrm{the}\;\mathrm{experimental}\;\mathrm{specimen}}-v_{\mathrm{organic}\;\mathrm{glass}}$$

The acoustic coupling phenomenon will affect the transmission of ultrasonic waves in the detected object and affect the detection result. Therefore, a coupling agent (honey was used in this paper) was used on the inspection surface to enhance the penetration ability of ultrasonic wave [38].

(3) Repeat steps (1) and (2) for a cycle after the single measurement. It is not until the completion of 35 cycles that it can be found that the concrete test block has produced significant erosion and cracks on the macro level.

(4) We used the AHP method to process ultrasonic nondestructive testing data, the processed Erd data and the MASS data into the computer, Erd database and mass database input ARIMA model. The ARIMA-Erd prediction model and ARIMA-Mass prediction model were constructed and run. Finally, the output variables of the prediction model were obtained. The superiority and statistical significance of the model were analyzed by AIC, BIC, ACF and PACF.

(5) MAE, MAPE, MSE and RMSE were used to evaluate the prediction accuracy of the experimental results, and the results obtained by the output variables after the model was established were analyzed and discussed.

## 3. Results

### 3.1. Mass Change

The mass was measured 35 times in the experiment, with the first 30 times for analysis and the last 5 times for predictive testing.

**Figure 4:**
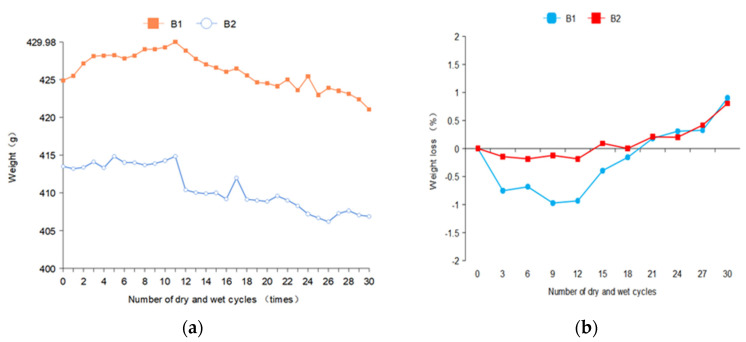
Mass changes of concrete specimens. (**a**) Mass loss curves of B1 and B2 concrete specimen (**b**) The mass loss rate curves of B1 and B2 concrete specimens were obtained.

In Figure 4, the mass of B1 and B2 shows a trend of a slow rise, then a trend of rapid decline after leveling off. After 30 dry–wet cycles, the mass loss rates of B1 and B2 were 0.901 and 0.805, respectively. This is due to the sulfate ions in the concrete after the reaction with the internal hydration products, resulting in erosion products in the pore accumulation, which fills the pores. In a certain period of time, the further reaction of the sulfate solution is blocked. The compactness of concrete is temporarily improved, so the mass is increased to a certain extent. However, after several dry–wet cycles, crystalline salt will be generated at the outer pore of the concrete specimen. The erosion products generated inside have expansionability, so the crystalline salt expansion stress will be generated, resulting in cracking and spallation of concrete, and the mass will be greatly reduced. The solution concentration of B2 was low and sulfate ions enter the concrete less, so the mass loss before the 12th time was less. In the later stage of erosion, the mass loss of concrete did not increase significantly due to the generation of erosion products and the shedding of aggregate. The mass of B2 significantly decreased after the 15th dry–wetting cycle because of the shedding of coarse aggregate.

### 3.2. Elastic Relative Dynamic Modulus

The combined ultrasonic speed of 35 dry–wet cycles tests were obtained, and the Erd were calculated.

**Figure 5:**
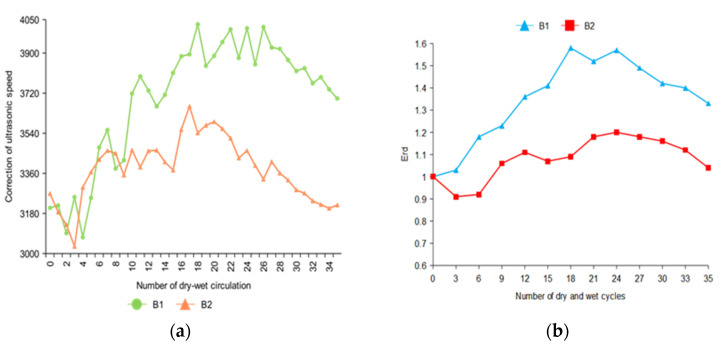
Changes in ultrasonic speed of concrete specimens after the correction of three-dimensional tomographic analysis. (**a**) Ultrasonic velocity loss curves of B1 and B2 concrete specimen (**b**) The ultrasonic velocity loss rate curves of B1 and B2 concrete specimens were obtained.

In Figure 5, the change of the ultrasonic speed of B2 was relatively gentle compared with that of B1 because B2 is immersed in 2% Na_2_SO_4_ solution. The corrosion of B2 was relatively slow, and the internal expansion product accumulation rate was also slow. When the specimen B1 was immersed in 10% Na_2_SO_4_, the change of ultrasonic speed was the most obvious.

Because of the different sulfate concentrations, the amount of sulfate ions in the solution that entered the concrete in the same time was different. The Erd of specimen B2 decreased in the first three cycles of drying and wetting. This was because the concentration of B2 erosion solution was low, and the generation rate of internal expansion products was less than the damage of the specimen in the soaking and drying cycles. Therefore, the Erd decreased accordingly. With the increase in the number of dry–wet cycles, the internal expansion product accumulated. The accumulated amount was greater than the damage amount of the soaking–drying cycles. After the descending stage, B1 and B2 showed an obvious upward trend due to the continuous accumulation of expansion products. After the 24th dry–wet cycle, the expansion products were generated in large quantities to produce an expansive force, and the internal compactness of concrete decreases. A large number of expansion products will also produce micro-cracks in the specimens, which will promote the degradation of concrete, resulting in the decline of Erd of B1 and B2.

### 3.3. Prediction Based on ARIMA Time Series

#### 3.3.1. Stationary Analysis of Time Series Data

**Figure 6:**
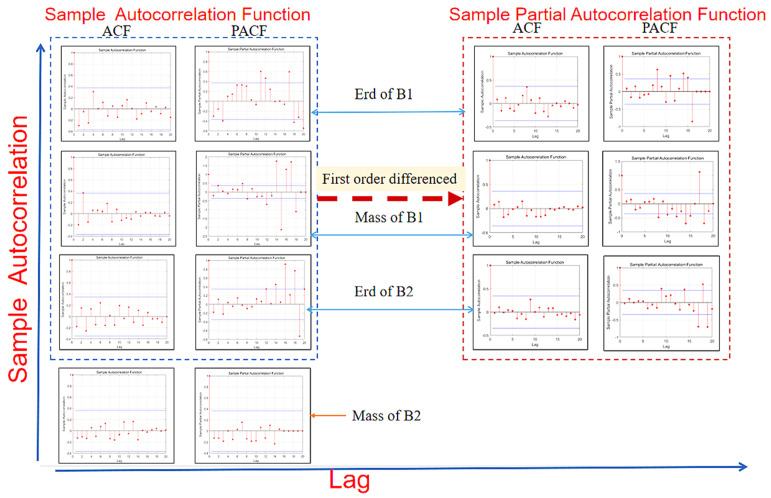
The initial data stability of B1 and B2 was tested by ACF and PACF test and post-difference test.

Figure 6 shows the ACF, PACF test and post-difference test for the initial data stability of B1 and B2. Before the first difference, it showed that the trailing of the PACF graph did not tend to 0, and the sharp value was too large. It was judged that the mass and Erd of B1 and the Erd of B2 belong to non-stationary time series data. Therefore, it was necessary to make a difference to them to maintain the stationarity of the time series of the ARIMA model. Then, *p* and *q* values of B1 and B2 ARIMA models were obtained by AIC and BIC ordering methods.

**Figure 7:**
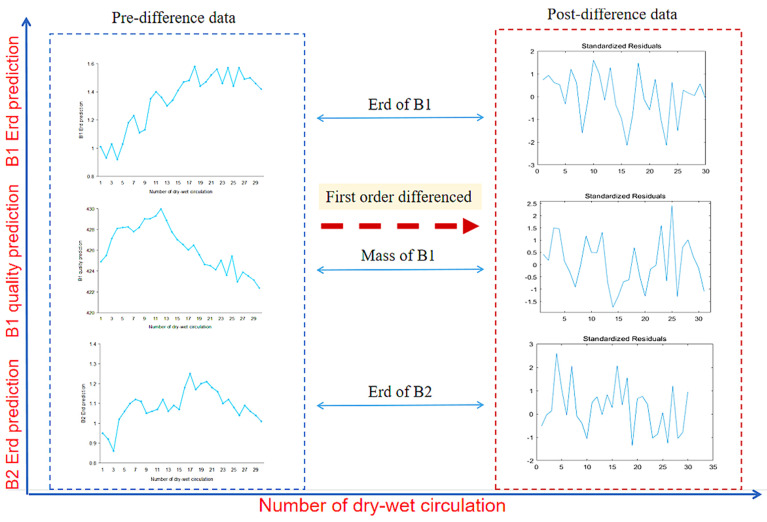
First-order difference of the ARIMA model.

Figure 7 shows the first-order difference graphs of ARIMA (2,1,1), ARIMA (5,1,3) and ARIMA (3,1,3). It can be seen that after the first-order difference, the trend of the original sequence (the trend must be non-stationary) is eliminated. The whole sequence basically oscillates around the determined mean value. Compared with the first-order difference, the second-order difference only enlarges the amplitude of the oscillation, so it is more appropriate to adopt the first-order difference for this sequence. In general, the first-order and second-order difference can make the sequence become stable.

In order to ensure that the order of the ARIMA model was consistent, ACF and PACF graphs were used to test the stationarity of the model again. After the difference of the model, it can be seen that the ACF autocorrelation functions tend to be 0 in the end. The number of sharp red sites in the PACF figure is greatly reduced, and the value is also decreasing. It proves that the model has become a stationary time series after the difference, and effective data has been input into the model.

By comparing the mass prediction and the Erd prediction, it can be found that the final trailing of the mass prediction tends to be 0. The Erd still tends to be slow after the difference. According to the stationary analysis, the mass prediction is selected as the better model for damage failure prediction.

#### 3.3.2. Stationary Analysis of Time Series Data

In order to check whether the ARIMA model conforms to the application of prediction, the residual test is carried out.

**Figure 8:**
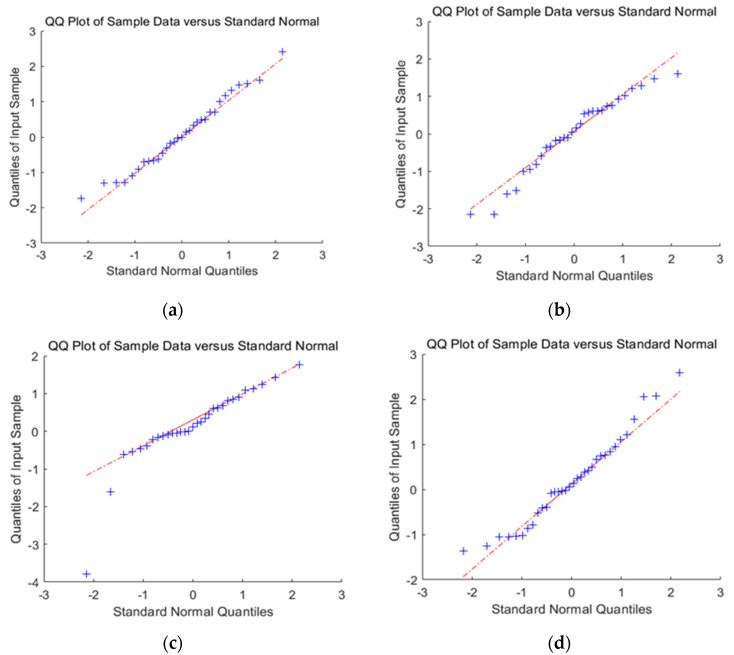
Erd of B1 and B2 and residual test of mass prediction model QQ-Plot; (**a**) QQ-plot of B1 mass prediction; (**b**) QQ-plot of Erd prediction of B1; (**c**) QQ-plot of B2 mass prediction; (**d**) QQ-plot of Erd prediction of B2.

In Figure 8, the linear graph of residual followed a linear relationship with the quantile, and all the sites were distributed near the line. Although the observed *P*-value of the late sites did not significantly exceed the expected value, they were close to the expected value. Therefore, the analysis model was reasonable. The D-W test shows that the D-W value of the two models is close to 2, so this model does not belong to the autocorrelation model.

When selecting mass prediction and relative motion prediction, it can be seen that the lower-left corners of the QQ-plots of mass prediction are the sites with low significance; that is, the sites that are not associated with traits are determined. The observed *P*-value of these sites should be consistent with the expected value. In the upper right corner of the graph are the sites with high significance, which are potential candidate sites associated with traits. These points are located above the diagonal; that is, the observed *P*-value of the site exceeds the expected value. This indicates that the effect of these sites exceeds the random effect, which, in turn, indicates that these sites are significantly correlated with the traits. This also means that the mass prediction represents a better model choice.

#### 3.3.3. Prediction Model Evaluation

**Figure 9:**
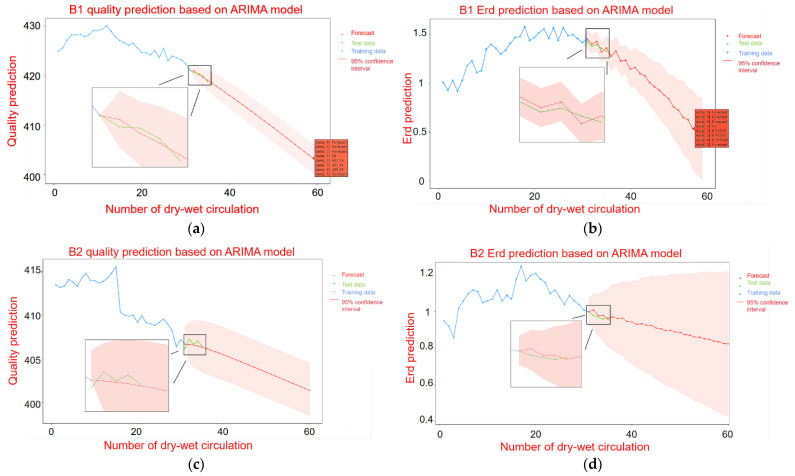
ARIMA time series model prediction of mass and Erd of B1 and B2; (**a**) B1 mass prediction; (**b**) Prediction of B1 Erd; (**c**) B2 mass prediction; (**d**) Prediction of B2 Erd.

Figure 9a,c show the mass prediction of B1 and B2 under the dry–wet cycle. Figure 9b,d show the Erd prediction of B1 and B2 under the dry–wet cycles. In Figure 9, the blue line represents the experimental training data, the green line represents the actual measurement data, and the red line represents the forecast trend data. The red line area represents the 95% confidence interval of the forecast data. According to Figure 9 and Table 3, since B1 and B2 adopt a first-order difference, the values of *p* and *q* are all less than 2; thus, the ARIMA model is a low-order model. It shows that the mass prediction model has an obvious trend of change. Therefore, the fluctuation of B1 in the mass prediction is small, and finally, it is transformed into a quasi-linear line. When the Erd prediction model adopts the first-order difference, the values of *p* and *q* are 5, and then the ARIMA model is a high-order model. This indicates that the change trend of Erd is not obvious, and there is obvious oscillation. Therefore, according to the previous experimental training data, the forecast curve is oscillating at the beginning and then smoothing out at the end.

Through the exercise of the previous experimental training data, the prediction curves of B1 and B2 were compared. It can be found that for both the mass prediction and the Erd prediction of B1 within 30 times, the Erd loss value of the specimen is 60% (mass loss is 5%). At this point, the sulfate attacks the concrete to the point of damage and failure. However, the prediction curve of B2 is slow, and the failure times of sulfate erosion loss are about 70 times. This is due to the different sulfate concentrations of B1 and B2, the erosion rate of B1 is obviously greater than that of B2, and the predicted failure times of B2 are also greater than that of B1.

According to the prediction results, the mass loss of B1 is predicted to reach 5% after the 59th dry–wet cycle to reach damage failure, and the mass is only 403.3 g. The Erd of elasticity decreased to less than 60% after the 53rd dry–wet cycle and reached loss failure, with a time span of 6 times. The mass prediction of B2 stops after the 103rd dry–wet cycle, and the mass loss reaches 5% to reach the loss failure. The Erd stops after the 101rd dry–wet cycle and drops below 60% to reach the loss failure. The time span is two times. Therefore, it can be mutually verified that the optimization effect of the model is good, and the prediction results are accurate. The difference in damage failure time between the two methods is not big.

Based on the mass of the ARIMA time series and the accuracy of the Erd prediction model were compared. The results of mass prediction and Erd prediction are given in Table 3.

According to Table 3 and Table 4, the MSE, MAE, RMSE and MAPE of the mass loss prediction and the Erd prediction are relatively small. It indicates that the deviation between the experimentally measured value and the predicted value of the ARIMA model is small. The optimization and prediction effect of the ARIMA model is good. By comparing the mass loss prediction with the Erd loss prediction, it can be found that the MSE, MAE and RMSE values of the mass loss prediction are all small. However, the MAPE value is obviously larger than the value of the Erd loss. This is because the mass value itself is large, and the fluctuation range is large, so the above situation occurs. For the QQ-Plot, ACF and PACF plots, it can also be judged that the mass loss prediction of sulfate erosion failure stability and optimization degree based on the ARIMA model is better than the Erd. In conclusion, the prediction accuracy and the results of the mass loss prediction of sulfate erosion failure based on the ARIMA time series model are better than those based on the Erd of the ARIMA time series.

The reason is that in the case of a small number of samples, the variation trend of mass loss prediction is simple and presents less volatility. The variation trend of Erd loss prediction is complex and has high volatility, which requires the development of a large number of optimized control parameters and a relatively large training database.

## 4. Conclusions

This paper introduces in detail the method of sulfate erosion loss failure of in-service tunnels combined with mass nondestructive testing, ultrasonic nondestructive testing and the ARIMA computer learning model. According to the proposed method, the study of sulfate erosion loss failure of concrete in-service tunnels can be divided into three stages. The first stage is to retain the corrosion inside the concrete of the service tunnel and conduct the dry–wet cycle sulfate erosion acceleration experiment. In the second stage, ultrasonic nondestructive testing and mass nondestructive testing are used to obtain the mass data and Erd data of concrete specimens, and the mass database and Erd database are established. In the third stage, ARIMA is used to establish the mass loss failure model and Erd loss failure model.

In this study, interesting results about sulfate loss failure of in-service tunnels are given:

1. Through the mass nondestructive testing and ultrasonic nondestructive testing of concrete specimens, we found that, with the increase in dry–wet cycles, the mass loss rate of the specimen showed a trend of decreasing slowly and then increasing rapidly after leveling off. The Erd decreases in the early stage due to the damage of the soaking–drying cycle, and increases rapidly in the later stage, then decreases slowly after leveling off.

2. Through the ARIMA model stability, residual test, MSE, MAE, RMSE and MAPE and other judgment basis, we can confirm that the ARIMA model can accurately predict the failure time of sulfate erosion of concrete in an in-service tunnel.

3. The proposed methods of computer learning and nondestructive testing can serve as prototypes for future practical applications. It has reference significance to judge the timely protection of the secondary lining of the tunnel in service and whether it needs to be dismantled.

## Figures and Tables

**Table 1:** Mixed proportion of concrete design of in-service tunnel.

Test Case Name	W/C	Water Consumption (kg·m^3^)	Gelled Material (kg·m^3^)	Aggregate (kg·m^3^)	28 Days Compressive Strength (MPa)	Concrete Grade
The ratio of the design	0.53	205	388	1813	39.5	C25

**Table 2:** Detection groundwater ion water samples at concrete borehole of tunnel.

Term	Ca^2+^ (mg/L)	Mg^2+^ (mg/L)	Na^+^ (mg/L)	SO_4_^2−^ (mg/L)	Cl^−^ (mg/L)	HCO_3_^−^ (mg/L)
ZK83 + 551	309.56	61.21	17.65	761.69	1.17	102.31
ZK83 + 509	317.15	66.53	15.54	699.33	1.18	116.90
ZK83 + 565	448.00	71.80	18.97	1020.04	1.20	122.77
ZK83 + 535	451.81	70.23	18.75	815.14	0.18	122.77
ZK83 + 523	419.86	68.68	16.44	824.05	1.18	116.92

**Table 3:** ARIMA time series prediction table (**a**) Predicted value and error analysis of time series B1 (**b**) Predicted value and error analysis of time series B2.

**(a)**				
**Dry–Wet Cycle**	**B1 Mass Measurement Value (g)**	**ARIMA Mass Prediction Value (g)**	**B1 Erd Measurement Value**	**Predicted Value of Erd of ARIMA**
31	421.07	421.03	1.43	1.45
32	420.46	420.84	1.38	1.40
33	420.4	420.14	1.40	1.43
34	419.85	419.63	1.36	1.32
35	418.74	419.02	1.33	1.36
B1 mass prediction: MAE = 0.236 MSE = 0.06808 RMSE = 0.2609 MAPE = 0.00281 B1 Erd prediction: MAE = 0.028 MSE = 0.00084 RMSE = 0.0290 MAPE = 0.1019				
**(b)**				
**Dry–Wet Cycle**	**B2 Mass Measurement Value (g)**	**ARIMA Mass Prediction Value (g)**	**B2 Erd Measurement Value**	**Predicted Value of Erd of ARIMA**
31	406.18	406.68	1.00	1.00
32	407.27	406.65	0.98	1.01
33	406.66	406.57	0.97	0.98
34	407.04	406.47	0.96	0.98
35	406.45	406.33	0.97	0.96
B2 mass prediction: MAE = 0.4 MSE = 0.19636 RMSE = 0.4431 MAPE = 0.00467 B2 Erd prediction: MAE = 0.014 MSE = 0.0003 RMSE = 0.0173 MAPE = 0.0721				

**Table 4:** ARIMA model was selected to predict the damage and failure of sulfate erosion specimens.

Specimen to Predict	ARIMA (*p*,*d*,*q*)	MSE	MAE	RMSE	MAPE	D–W	Loss of Failure
B1 Mass Prediction	(2,1,1)	0.06809	0.236	0.2609	0.00281	1.7824	59 times (403.3 g)
Prediction of of B1 Erd	(5,1,3)	0.00084	0.028	0.0290	0.1019	1.8081	53 times (0.535)
B2 Mass Prediction	(1,0,1)	0.19636	0.4	0.4431	0.00467	2.2272	103 times (392.65 g)
Prediction of of B2 Erd	(3,1,3)	0.0003	0.014	0.01732	0.0721	1.9091	101 times (0.596)

## Data Availability

The data presented in this study are available on request from the corresponding author.
